# Supplementary material for: Robust skeletal motion tracking using temporal and spatial synchronization of two video streams
Source: PLoS One. 2025 Aug 7;20(8):e0328969. doi: 10.1371/journal.pone.0328969 (PMC12331063; doi:10.1371/journal.pone.0328969)
Supplement: Attachment — Submitted filename: answer_reviewers.pdf [file pone.0328969.s001.pdf]

Journal Requirements:

When submitting your revision, we need you to address these additional requirements.

1. Please ensure that your manuscript meets PLOS ONE's style requirements, including those for file naming. The PLOS ONE style templates can be found at

https://journals.plos.org/plosone/s/file?id=wjVg/PLOSOne_formatting_sample_main_body.pdf and

https://journals.plos.org/plosone/s/file?id=ba62/PLOSOne_formatting_sample_title_authors_affiliations.pdf

2. Please note that PLOS ONE has specific guidelines on code sharing for submissions in which author-generated code underpins the findings in the manuscript. In these cases, we expect all author-generated code to be made available without restrictions upon publication of the work. Please review our guidelines at https://journals.plos.org/plosone/s/materials-and-software-sharing#loc-sharing-code and ensure that your code is shared in a way that follows best practice and facilitates reproducibility and reuse.

3. Thank you for stating the following financial disclosure: [This project has received funding from the Research Council of Lithuania (LMTLT), agreement No S-PD-24-29.].

Please state what role the funders took in the study. If the funders had no role, please state: ""The funders had no role in study design, data collection and analysis, decision to publish, or preparation of the manuscript.""

If this statement is not correct you must amend it as needed.

Please include this amended Role of Funder statement in your cover letter; we will change the online submission form on your behalf.

4. Thank you for stating the following in the Acknowledgments Section of your manuscript: [This project has received funding from the Research Council of Lithuania (LMTLT), 461

agreement No. S-PD-24-29.]

We note that you have provided funding information that is not currently declared in your Funding Statement. However, funding information should not appear in the Acknowledgments section or other areas of your manuscript. We will only publish funding information present in the Funding Statement section of the online submission form.

Please remove any funding-related text from the manuscript and let us know how you would like to update your Funding Statement. Currently, your Funding Statement reads as follows:

Please include your amended statements within your cover letter; we will change the online submission form on your behalf.

5. Please include your full ethics statement in the 'Methods' section of your manuscript file. In your statement, please include the full name of the IRB or ethics committee who approved or waived your study, as well as whether or not you obtained informed written or verbal consent. If consent was waived for your study, please include this information in your statement as well.

Answer.

Thank you for investing your time and attention in handling our submission. We have carefully considered every point raised in the decision letter and the accompanying reviews, and we have revised the manuscript accordingly: our methodological distinctions, refining the figures for grayscale accessibility, expanding the Results and Discussion to make our contributions to upper-limb rehabilitation unmistakable. We believe these edits fully address the reviewers and your recommendations and improve the clarity and impact of the work.

We have reviewed the requirements, and made small adjustments over all manuscript, especially about the affiliations section. We have included additional information about the funding. Indeed, the funding status matches the statement: *""The funders had no role in study design, data collection and analysis, decision to publish, or preparation of the manuscript."".* Also, we have included this information in cover letter. And we would like to request the editor, to include funding information in the official Funding Statement with the manuscript. Moreover, full ethics statement is now included in the Methods section.

Reviewer #1: The paper presents a novel dual-camera skeletal tracking system aimed at improving depth accuracy in human pose estimation for rehabilitation monitoring. Positioned within the context of low-cost, real-time rehabilitation applications, its main contribution is the integration of temporal and spatial synchronization, a linear regression-based depth error correction model, and Kalman filtering to enhance robustness and accuracy.

While multi-camera setups are not new, the focus on dynamic depth error correction via linear regression, combined with Kalman filtering, offers meaningful innovation. The work is well-motivated, addressing a critical barrier to affordable home-based skeletal tracking.

The paper details the synchronization, regression correction, and Kalman filtering with clear mathematical formulations. However, some aspects need clarification:

(1) the linear regression model may oversimplify complex 3D depth errors; exploring or justifying more sophisticated models would strengthen the approach;

Answer. We agree that our linear model may underfit inherently non-linear joint dynamics. We believe, that sophisticated models would require additional research, since the processing time could influence systems capabilities for deployment. We have updated the Discussion to acknowledge this limitation explicitly and to outline lightweight non-linear alternatives—such as Gaussian-process regression, kernel ridge regression, and compact neural models, as promising avenues for future work. However,

(2) calibration and alignment assumptions are critical but only briefly discussed; deeper elaboration on calibration errors and mitigation would aid reproducibility. The methodology is sound but would benefit from a more thorough discussion of limitations.

Answer. We appreciate the reviewer's insightful comment. Indeed, in our study, we intentionally employed a straightforward calibration and alignment procedure with only minimal manual intervention. We aimed to experiment with a simple dual-camera setup that does not rely on checkboards or other similar solutions. Methods section now clarifies this, and includes a little more detail on the setup, as following:

Both cameras were mounted on tripods at approximately the same height (±1 cm) and oriented to form a nominal 90° angle. We approximate each camera as an ideal pinhole (principal point in the center of the image, no distortion coefficients) using the manufacturer's horizontal field of view to infer the focal length in pixels.

While acknowledging that this simplicity introduces inherent calibration limitations and should be further discussed. Essentially, it significantly enhances the reproducibility and practical applicability of our approach, particularly in scenarios such as variable user profiles, lightening conditions might impede usability. Thus, we have modified and extended discussion section as following:

Despite promising results, certain factors limit its accuracy. Different subject profiles, such as children, frail elderly people, or orthotic wearers, can distort limb proportions and cause pose errors. Secondly, fluctuating illumination or cluttered backgrounds may suppress key-point confidence and could bias depth estimation. Our configuration assumes the ideal 90 angle between cameras, precise orientation and height consistency (±1cm), any actual deviation or incomplete perspective distortion may lead to systematic errors. Furthermore, our synchronization method mainly compensates for rotations around the Y-axis and corrects potential out-of-plane rotations with insufficient compensation. Unmodeled image blurs can also affect the accuracy of joint detection and depth estimate. Therefore, future work could consider using explicit calibration methods with established methods (such as Zhang's method or fiducial marker-based calibration like AprilTags to mitigate distortion and alignment errors. However, our simplified approach is practical and emphasizes the ease of reproducibility and deployment, especially for clinical or home rehabilitation.

Furthermore, performing intrinsic camera calibration under simplified assumptions can introduce inaccuracies due to inherent lens distortion effects (specifically radial and tangential distortions). Thus, inferring focal length from the manufacturer's stated horizontal field of view and image width contributes to additional error. Small discrepancies between the nominal field of view and the actual in-scene focal length can result in scale errors of up to 5%. Those scale errors are likely directly propagating into reconstructed 3D joint coordinates, causing consistent underestimation or overestimation of depth across the entire skeleton. Thus, any unknown focal length mismatch remains uncorrected, which may be particularly problematic when subjects perform exercises at varying distances from the cameras.

Experiments are well-documented, with both quantitative metrics and qualitative visualizations demonstrating superior depth estimation accuracy over baseline methods. Still, improvements are suggested:

(1) include more diverse baselines (e.g., recent transformer-based models);

Answer. We appreciate this suggestion and have expanded the introduction and results to include two more transformer-based approaches, along to existing transformer-based methods: Hourglass Tokenizer (HoT) and MotionAGFormer. As we have included in the manuscript, those papers report lower MPJPE values than ours. However, we have conducted a direct empirical comparison against a similar transformer-based method SlowFastFormer (2024). As results show our dual-camera pipeline achieves lower elbow and wrist level errors than SlowFastFormer while running in real time.

(2) add ablation studies to isolate the contributions of each component (e.g., regression vs. Kalman filtering).

Answer. We appreciate this suggestion and have added a dedicated ablation study that quantifies the impact of every algorithmic block on both accuracy and inference time. Because several modules are functionally indispensable, the outright removal would break the processing chain. Therefore, we have replaced each one with the simplest viable alternative. For example, Kalman filter was disabled, and any missing joint was filled by the most recent valid estimate; regarding linear regression, the network depth output was directly fed to the fusion stage, and instead of spatial and temporal synchronization we aligned frames solely by the closest time-stamp.

A discussion of practical applications and integration with pose refinement algorithms, such as:

- http://dx.doi.org/10.48550/arXiv.2112.13715
- https://doi.org/10.1109/LSP.2023.3295756
- https://doi.org/10.1016/j.sigpro.2024.109598

would further enhance the paper.

Answer. We appreciate the reviewer's suggestion to discuss integration with pose-refinement methods. In practice, our pipeline can be paired with a lightweight, learned refinement modules. And most of the proposed post-processing modules are suitable for real-time applications like home rehabilitation monitoring, sports analytics or similar. We have updated the discussion section accordingly:

In practical settings, such as clinical rehabilitation monitoring, sports biomechanics, and human-computer interaction, pose estimates may suffer from temporal jitters and occasional large errors (e.g., during occlusions or rare poses). By integrating pose refinement algorithms like SmoothNet, FLK, or HiMoReNet, to our proposed dual-camera pipeline, could offer a refined the smoothness and accuracy of skeleton trajectories. Such post-processing modules usually are designed as a 'plug--and--play' as input takes a sequence of 2D/3D joint coordinates and output smoothed poses by modeling long-range temporal relations per joint. For example, SmoothNet can be applied to each joint channel individually, which is particularly

appealing in rehabilitation context, although additional refinements would be needed for real-time applications. On the other hand, FLK combines an adaptive Kalman filter with a biomechanical constraint adjustment and a low-pass filter, and offers a real-time join refinement solution for a desktop PC. HiMoReNet uses a hierarchical architecture that group joints and captures long-range temporal context, and then refines each group's motion while leveraging global body interactions. Thus, using the HiMoReNet module in our proposed pipeline could enforce biomechanically plausible trajectories, which is crucial, if the full system would be applied for montion quantification or anomaly detection.

Reviewer #2: The manuscript presents a well-organized and timely contribution to the field of skeletal motion tracking, particularly targeting rehabilitation scenarios. The integration of a dual-camera system, along with spatial and temporal synchronization mechanisms such as linear regression-based depth correction and Kalman filtering, reflects a thoughtful approach to addressing known limitations of single-camera systems. The proposed method is validated across custom datasets and benchmarked against established models, showing promising improvements in depth accuracy. The clarity of presentation and the thorough methodology are commendable. The work holds strong potential for applications in telerehabilitation and home-based monitoring. However, there are several areas where the manuscript could be further strengthened:

1. While the paper introduces a dual-camera system, the novelty is somewhat incremental given existing literature on stereo vision and multi-view pose estimation. The manuscript would benefit from a clearer articulation of how the presented system differs fundamentally in architecture, methodology, or application domain from prior approaches.

Answer. Thank you for pointing out the need for clearer differentiation from prior stereo-vision work. In the revised manuscript we now emphasize our contribution in more clear way. There is an updated closing paragraph in the Introduction section. We have also specified, that our main experiments point to upper limb activities (as you pointed out in further comments). Moreover, an expanded Discussion section contrasts these elements with state-of-the-art multi-view methods and existing limitations are discussed and displayed.

2. The real-time applicability of the system is emphasized, yet there is no quantitative evidence provided to substantiate this claim. Details on processing time per frame, latency, and computational requirements should be reported, especially considering the use of multiple video streams and filtering algorithms.

Answer. Thank you for a really important suggestion. We have added a performance analysis that reports inference time on a desktop GPU. The complete pipeline processes each frame well below the 33 ms. Moreover, we have included a small ablation that quantifies the impact of every algorithmic block on both accuracy and inference time. Because several modules are functionally indispensable, the outright removal would break the processing chain. Therefore, we have replaced each one with the simplest viable alternative. For example, Kalman filter was disabled, and any missing joint was filled by the most recent valid estimate; regarding linear regression, the network depth output was directly fed to the fusion stage, and instead of spatial and temporal synchronization we aligned frames solely by the closest time-stamp.

3. Discuss on how the system would generalize to broader scenarios—such as diverse subject profiles, variable lighting, or real-world rehabilitation environments.

Answer. Thank you for prompting us to elaborate on generalization issues. We have expanded the Discussion with the following text:

Despite promising results, certain factors limit its accuracy. Different subject profiles, such as children, frail elderly people, or orthotic wearers, can distort limb proportions and cause pose errors. Secondly, fluctuating illumination or cluttered backgrounds may suppress key-point confidence and could bias depth estimation. Our configuration assumes the ideal 90 angle between cameras, precise orientation and height consistency 1 cm, any actual deviation or incomplete perspective distortion may lead to systematic errors. Furthermore, our synchronization method mainly compensates for rotations around the Y-axis and corrects potential out-of-plane rotations with insufficient compensation. Unmodeled image blurs can also affect the accuracy of joint detection and depth estimate. Therefore, future work could consider using explicit calibration methods with established methods (such as Zhang's method or fiducial marker-based calibration like AprilTags to mitigate distortion and alignment errors. However, our simplified approach is practical and emphasizes the ease of reproducibility and deployment, especially for clinical or home rehabilitation.

4. The Kalman filter implementation is well described mathematically, but the choice and tuning of parameters (e.g., noise covariance matrices) are not discussed. Providing this information is essential for reproducibility and for understanding the system's adaptability across different motion patterns.

Answer. Thank you for the comment, we have included more details regarding the choice and tunning of initial parameters, equations 13-15. The inclusions are highlighted in the manuscript.

5. The Pareto optimization technique used for temporal alignment is innovative but lacks intuitive explanation. A visual diagram or step-by-step example would be beneficial, particularly for readers from non-engineering backgrounds.

Answer. Thank you for this comment. We have included a flowchart diagram to provide a more intuitive understanding for the readers. We have included a reference about the Pareto optimization, and the details required to reproduce our work. However, we feel that a step-by-step example may be not entirely appropriate for this particular study. We hope, that this is enough to satisfy this comment of the reviewer. We have also included a short paragraph in discussions regarding the trade-off of Pareto optimization.

The Pareto optimization technique helps balance two competing goals: making joint positions from two camera streams line up accurately (synchronization), and keeping processing time or resource use low (computational cost).

We begin by defining a discrete set of possible time shifts for each stream (e.g., moving video forward or backward by a few frames). For every combination of time shifts, we evaluate how well the joint positions match in depth (Z-axis) and how expensive it is to implement the shift in terms of resources. These pairs of scores, accuracy and cost, are recorded for all combinations of shifts.

The next step is Pareto filtering, where we eliminate all "dominated" solutions, those that are worse in both accuracy and cost compared to some other solution. The remaining set forms the Pareto front: the most efficient trade-offs.

The final time adjustment is chosen based on the application needs. If precise joint alignment is crucial (e.g. in medical analysis), we select the most accurate solution. If low latency is key (e.g., in real-time systems), we prioritize low cost. If both matter, we can use a weighted score to find the best balance.

6. Although comparisons with state-of-the-art models like PoseFormer and SlowFastFormer are presented, it is not fully clear if these baselines were evaluated under the same dataset and preprocessing conditions. Greater transparency in the benchmarking methodology is recommended.

Answer. Thank you for the comment, it is a very good point. It is not easy to benchmark other models in dual camera setup. Initially we did consider running only preprocessing of such models (2D pose estimation) on both camera feeds and then triangulating the 3D coordinates. However, for this work, we ultimately decided to use a single frontal camera setup for our 3D pose estimation algorithms. Now, we have included this information in the manuscript. Moreover, we have also included a short paragraph about this limitation in discussion section.

7. The claim of robustness against occlusion is supported in theory, but the experimental validation is somewhat limited in this regard. Additional test cases with realistic occlusions (e.g., arms crossing the body, partial visibility) would help validate this important aspect.

Answer. We thank the reviewer for emphasizing the need to clarify our occlusion claims. As shown in Table 8, partial self-occlusion scenarios (e.g., arms crossing the torso) were investigated. In response, we have enriched the Discussion with a deeper analysis of these results and explicitly acknowledged the limited scope of our occlusion tests, which were focused on upper-body joint visibility. Statements about occlusion

robustness have been revised to accurately reflect the experimental conditions and their constraints.

8. Depth correction is achieved using a linear regression model, which may be effective in structured tasks but could lack flexibility in more dynamic or non-linear motion scenarios. Exploring the potential of non-linear regression techniques could be valuable.

Answer. We agree that our linear model may underfit inherently non-linear joint dynamics. We have updated the Discussion to acknowledge this limitation explicitly and to outline lightweight non-linear alternatives—such as Gaussian-process regression, kernel ridge regression, and compact neural models, as promising avenues for future work.

9. Visualizations are helpful but could be refined for accessibility. The color-coded skeletons are difficult to distinguish when printed in grayscale. Including markers or labels could improve figure readability across different formats.

Answer. Thank you for noting the grayscale-accessibility issue. We have remade skeleton visualisations so that joint markers are now hollow circles and the limb connections use solid dark lines for the front view and lighter grey lines for the side view. These tone and shape differences should remain distinguishable when the figures are printed in black-and-white or viewed by readers with colour-vision deficiencies. Manuscript text have been updated to explain the new coding.

10. Some redundancy is observed in the text, particularly in repeated phrases describing depth distortion patterns. Condensing these sections would improve flow without diminishing clarity.

Answer. Thank you for your valuable feedback. Our revised manuscript now includes restructured, and edited the Results section to improve its readability and flow. This involved shorter paragraphs, cleaner and more condensed descriptions of figures, consolidating descriptions of similar results flow, and little refining of the language. As we looked into the manuscript, we believe, that mostly results section needed such improvement. However, other parts of the manuscript text also had small edits.

11. The manuscript frames the work within a rehabilitation context, yet it lacks discussion on how this system could be integrated with clinical protocols or validated using standardized outcome measures. Even a brief exploration of future clinical validation would increase the paper's practical relevance.

Answer: We thank the reviewer for this valuable and constructive comment. We agree that discussing potential clinical validation and integration with rehabilitation protocols is essential for demonstrating the practical relevance of our work. In response, we have supplemented the Discussion section (after line 438) to clarify the type of rehabilitation movements studied. We also added a paragraph outlining directions for future validation using standardized outcome measures, including the Motor Assessment Scale (MAS) and the Disabilities of the Arm, Shoulder, and Hand (DASH) questionnaire. Furthermore, we propose conducting pilot studies with patient cohorts to evaluate the system's integration into clinical workflows and to gather usability feedback. We believe these additions directly address the reviewer's concerns and strengthen the manuscript's clinical relevance.

12. The scope of the method appears limited to upper-limb motion, but the paper does not address whether this framework can be extended to full-body or lower-limb activities. A short note on system scalability would provide helpful context for broader application.

Answer: We appreciate the reviewer's helpful comment. As correctly noted, the current scope of our study is limited to upper-limb motion, focusing on exercises such as arm adduction and flexion (as shown in Table 2). We have reviewed and edited all parts of the paper (title, abstract, results, discussions and conclusions) to correctly reflect the scope of the presented method. We have now included a note in the Discussion section clarifying that the system is inherently scalable to full-body or lower-limb motion tracking. With

appropriate adjustments to camera setup and calibration, the framework can support broader rehabilitation applications, which we plan to explore in future studies.
